# Evaluation and Validation of a Real-Time PCR Assay for Detection and Quantitation of Human Adenovirus 14 from Clinical Samples

**DOI:** 10.1371/journal.pone.0007081

**Published:** 2009-09-17

**Authors:** David Metzgar, Greg Skochko, Carl Gibbins, Nolan Hudson, Lisa Lott, Morris S. Jones

**Affiliations:** 1 Department of Respiratory Disease Research, Naval Health Research Center, San Diego, California, United States of America; 2 Clinical Investigation Facility, David Grant United States Air Force (USAF) Medical Center, Travis, California, United States of America; 3 Advanced Diagnostic Laboratory, Lackland Air Force Base (AFB), Texas, United States of America; Institut Pasteur Korea, Republic of Korea

## Abstract

In 2007, the Centers for Disease Control and Prevention (CDC) reported that Human adenovirus type 14 (HAdV-14) infected 106 military personnel and was responsible for the death of one U.S. soldier at Lackland Air Force Base in Texas. Identification of the responsible adenovirus, which had not previously been seen in North America and for which rapid diagnostic tools were unavailable, required retrospective analysis at reference laboratories. Initial quarantine measures were also reliant on relatively slow traditional PCR analysis at other locations. To address this problem, we developed a real-time PCR assay that detects a 225 base pair sequence in the HAdV-14a hexon gene. Fifty-one oropharyngeal swab specimens from the Naval Health Research Center, San Diego, CA and Advanced Diagnostic Laboratory, Lackland AFB, TX were used to validate the new assay. The described assay detected eight of eight and 19 of 19 confirmed HAdV-14a clinical isolates in two separate cohorts from respiratory disease outbreaks. The real-time PCR assay had a wide dynamic range, detecting from 10^2^ to 10^7^ copies of genomic DNA per reaction. The assay did not cross-react with other adenoviruses, influenza, respiratory syncytial virus, or common respiratory tract bacteria. The described assay is easy to use, sensitive and specific for HAdV-14a in clinical throat swab specimens, and very rapid since turnaround time is less than four hours to obtain an answer.

## Introduction

Human adenoviruses (HAdVs) were the first respiratory viruses to be isolated and characterized. They were initially isolated from cultured adenoid tissue, within which these viruses may remain viable in a quiescent and asymptomatic state [1]. HAdVs were first identified as agents of disease among military trainees suffering from epidemics of acute respiratory disease (ARD), in the early 1950s [2]. Epidemiological studies confirmed and continue to show that adenoviruses are the primary cause of ARD epidemics among military recruits [3], [4] and also a common cause of both endemic and epidemic ARD among civilians. The diverse human pathogens of the genus *Mastadenovirus* in the family Adenoviridae are categorized into 53 types. These viruses have been grouped into seven species (A–G), based on their immunochemical responses, nucleic acid characteristics, hexon and fiber protein characteristics, biological properties, and phylogenetic relationships [5], [6]. They are transmitted by the fecal-oral route and the respiratory route and are associated with acute respiratory disease (ARD), conjunctivitis, genitourinary infections, and gastroenteritis [7].

Although basic military trainee sites can involve several types, public health response measures at military facilities are weakened because detection of ARD causing HAdVs is primarily accomplished by conventional PCR [8]. Primarily these infections are caused by HAdV-4 and –7 [8]; although HAdV-3, -11, -14, and -21 have also been detected at military bases and civilian settings as well [8], [9], [10]. Unfortunately, in February 2007, when a HAdV-14 febrile respiratory disease outbreak occurred in basic military trainees at Lackland Air Force Base [11], it took nearly three months to determine which genotype was responsible for infection [11]. Because adenovirus types vary in terms of clinical manifestation, outbreak severity, and susceptibility to developed vaccines, it is very useful to know which HAdV type is involved in any specific case or outbreak. Since the initial HAdV-14 outbreak in North America, it has been shown that the outbreak strain only contains one deletion in the fiber gene (ΔK250–E251) which differentiates it from the prototype deWit strain (HAdV-14p) and has been provisionally named HAdV-14a [12].

The J.B.A.I.D.S. thermocycler was conceived and developed to standardize biowarfare threat agent detection equipment to meet the need within the Department of Defense to enhance protection of U.S. military personnel in deployed locations. The software used in the J.B.A.I.D.S. is similar to the software used in the LightCycler 2.0. The thermocycler is a ruggedized and portable biological agent identification system that is capable of simultaneously identifying multiple biological agents.

In this study we developed a reliable, rapid real-time PCR assay that detects a 225 bp segment of the hexon gene of both the HAdV-14p and -14a genomes on the J.B.A.I.D.S. and LightCycler 2.0 thermocycler platforms, and validated the assay using both defined preparations of isolated virus and original clinical throat swabs from recognized HAdV-14 outbreaks in military populations.

## Results

### Detection of clinical isolates

**Figure 1:**
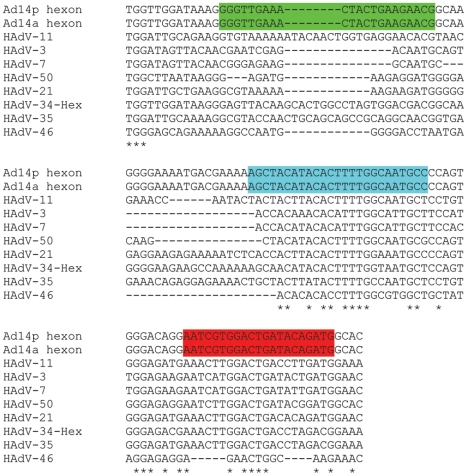
Alignment of species HAdV-B Hexon sequences which span the HAdV-14 primers and TaqMan probe. Note that the HAdV-14a and HAdV-14p sequences are identical. The forward primer sequence is highlighted in green, the FAM/TAMRA probe is highlighted in aqua, and the reverse primer is highlighted in red. Asterisks underneath sequences highlight identity in the species.

Our real-time J.B.A.I.D.S. PCR assay detects a unique 225 bp segment in the hexon gene of the HAdV-14p and -14a genomes (Fig. 1). This region is unique since there is little sequence homology in this region. The detection of HAdV-14a by conventional and real-time LC PCR of the clinical isolates is summarized in Table 1. We tested 27 HAdV-14-positive clinical samples from the Naval Health Research Center (NHRC) and the Advanced Diagnostic Laboratory (ADL) as well as 24 HAdV-14-negative clinical specimens from patients with ARD in similar populations (Tables 1 and 2). These samples were scored negative if no amplicon was acquired through conventional PCR for HAdV-3, -4, -7, -11, -14, or –21. All of these samples were correctly identified by the described assay. These results, generated at two separate institutions, support appropriate sensitivity of the assay and demonstrate that it does not generate false positive results from human genomic sequence or common commensals occurring in throat swabs (Tables 1 and 2). Therefore, our results were 100% concordant with original results generated with well-validated standard PCR and sequence-based assays [13].

**Table 1:** Summary of Human adenovirus 14 detection by conventional and real-time PCR in clinical specimens from the NHRC.

	Conventional PCR result from NHRC		J.B.A.I.D.S. real-time PCR for Adenovirus 14		LightCycler real-time PCR for Adenovirus 14	
	Positive	Negative	Positive	Negative	Positive	Negative
HAdV-14 positive	19	0	19	0	19	0
HAdV-14 negative	0	20	0	20	0	20

**Table 2:** Summary of Human adenovirus 14 detection by conventional and real-time PCR in clinical specimens from the ADL.

		J.B.A.I.D.S. real-time PCR for Adenovirus 14	
		Positive	Negative
Conventional PCR result from ADL	HAdV-14 positive	8	0
	HAdV-14 negative	0	4

### Sensitivity of HAdV-14 assay

**Figure 2:**
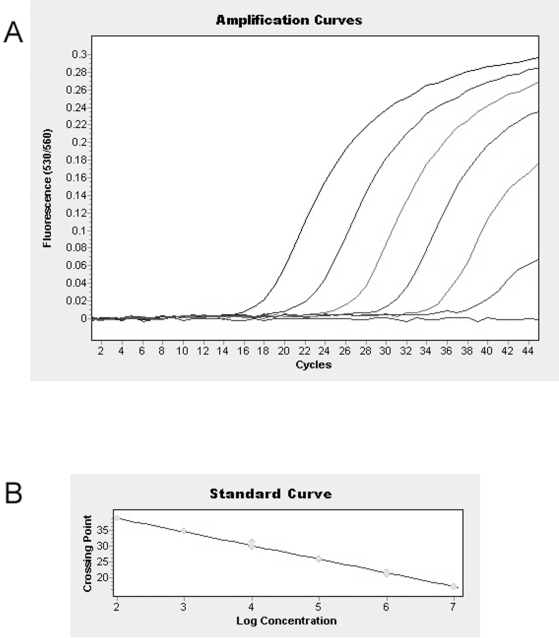
Sensitivity of Human adenovirus 14 detection by real-time J.B.A.I.D.S. PCR in a series of 10-fold dilutions of genomic HAdV-14 DNA (10^2^ to 10^7^) per PCR reaction. (A) Amplification profile of real-time J.B.A.I.D.S. PCR. (B) Linear regression of the standard curve.

A standard curve was created using 10-fold dilutions with genomic HAdV-14 DNA (10^2^ to 10^7^ genome copies per assay) from the deWit strain (accession # AY803294) to allow determination of the concentration of adenovirus in the clinical samples (Fig. 2). The lower limit of detection for the assay was 100 genomic copies per reaction (Fig. 2). Linear regression of the *CP* values and the quantity of genomic DNA revealed a negative linearity (error  = 0.05 and a slope -3.76) corresponding to an 84.5% PCR efficiency. The dynamic range for the assay was 10^2^ to 1.0×10^7^ HAdV-14 DNA genomic copies per PCR reaction. This experiment was repeated twice and yielded similar results. The viral load in analyzed throat swab samples varied from less than 4×10^5^ to 2.1×10^8^ genomic copies per milliliter (Table 3).

**Table 3:** Range of viral load in NHRC throat swabs.

Samples	Viral load genomic copies/ml of throat swabs
1	1.8×10^6^
2	5.8×10^6^
3	2.7×10^6^
4	2.6×10^6^
5	6.3×10^7^
6	8.9×10^7^
7	1.0×10^6^
8	3.0×10^7^
9	6.7×10^6^
10	1.0×10^7^
11	1.1×10^7^
12	6.8×10^6^
13	1.0×10^8^
14	2.1×10^8^
15	4.9×10^6^
16	1.2×10^7^
17	<4×10^5^
18	3.3×10^6^
19	7.4×10^5^

### Specificity of HAdV-14 assay

Because of the high degree of genetic similarity between the HAdV types within species HAdV-B, we wanted to demonstrate that our assay was specific to HAdV-14. The assay did not generate amplicons from other adenovirus genotypes that cause respiratory disease (Table 4). Furthermore, the assay was unable to generate an amplicon from genomic DNA extracted from bacterial and/or viral agents known to cause respiratory disease or to reside in the respiratory tract (Table 4).

**Table 4:** Specificity table of HAdV-14 assay.

Pathogen	HAdV-14 assay
HAdV-3	–
HAdV-4	–
HAdV-7	–
HAdV-11	–
HAdV-21	–
*Haemophilus influenzae*	–
*Influenza A virus*	–
*Human rhinovirus*	–
*Human parainfluenza virus*	–
*Human respiratory syncytial virus*	–
*Chlamydophila pneumoniae*	–
*Escherichia Coli*	–
*Klebsiella pneumonia*	–
*Pseudomonas aeruginosa*	–
*Mycoplasma pneumoniae*	–
*Legionella Pneumophila*	–

One nanogram of genomic DNA was used as a DNA template in each assay. Assays were performed in triplicate. Samples were negative at 45 cycles of amplification.

## Discussion

There are several studies describing adenovirus detection [14]–[19]. However, there are no known type-specific real-time PCR assays to detect HAdV-14a. This is the first study demonstrating a sensitive and specific real-time PCR assay that detects HAdV-14 in clinical samples. Our assay detected a 225-bp amplicon in the hexon gene of the HAdV-14 genome in samples from patients who tested positive for HAdV-14 via conventional PCR.

In 2006 and 2007 HAdV-14 emerged as a significant contributor to acute respiratory disease (ARD) and severe pneumonias in several US cities and at military recruit training centers across the United States [8], [11], [12], [20]. HAdV-14a outbreaks can cause severe pneumonia and occasional death in patients of all ages, as evidenced by the HAdV-14a outbreaks at Lackland AFB between February and July of 2007 and the temporally associated outbreaks among civilians in Oregon [11]. It is important to have diagnostic tools readily available that can determine which adenovirus is causing a particular outbreak. Although this assay works well on the LightCycler 2.0, it was important that this assay also worked well on the J.B.A.I.D.S. system since this platform is utilized at U.S. military installations in the United States as well as overseas. Further studies should be done to generate multiplex assays that detect all common epidemic respiratory adenoviruses.

Interestingly, the viral loads for HAdV-14 that we detected in uncultured clinical specimens were 1 to 3 logs below those seen in our lab for other common types, including HAdV-3, -4, -7, and –21 (data not shown). If the patient samples were acquired after peak viremia, this could explain the lower viral loads. Another possibility is that HAdV-14a may not be very effective at replicating due to unique genetic characteristics. This offers further purpose for the development of real-time assays, as these assays are generally more sensitive than standard PCR assays or rapid antigen tests [21], [22]. Having the capability to quantitate viral loads will be a valuable tool to further our understanding of type-specific aspects of adenoviral disease.

Including nucleic acid extraction, this assay can be used to determine whether or not a sample is positive or negative in less than four hours. Furthermore, the material cost of each test is 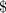
2.16. This real-time PCR assay demonstrated 100% concordance with the conventional PCR assays [13] initially used to identify HAdV-14 in these samples and did not generate any false negatives or false positives. This assay provides quantitative data and does not cross react with other ARD-causing adenoviruses. Thus, if another HAdV-14 outbreak occurs within the Department of Defense or within the civilian community, similar to the outbreak at Lackland AFB, we will have the tools available to rapidly detect HAdV-14 at low cost.

In conclusion, we generated a cost effective real-time PCR assay optimized for the J.B.A.I.D.S. and LightCycler 2.0 platforms, and demonstrated that this assay is sensitive and specific for HAdV-14. If accredited as an in-house diagnostic test in labs serving affected populations, this assay will be useful as a routine diagnostic tool for HAdV-14 infection.

## Materials & Methods

### Clinical samples

Informed consent was obtained in writing. The IRB-approved inclusion criteria of subjects enrolled at Lackland AFB were individuals greater than 18 years of age who presented with upper respiratory illness symptoms to the Wilford Hall Medical Center Hospital or clinics and military trainees who were 18 years or older at the time of enrollment who presented to recruit health clinics with URI symptoms or pneumonia. IRB-approved inclusion criteria for consented subjects enrolled through the NHRC febrile respiratory illness surveillance system were military recruits reporting for medical care with respiratory symptoms and a fever of ≥38°C. Samples collected through the NHRC program were oropharyngeal swabs resuspended in COPAN Viral Transport Medium (VTM), and subsequently frozen at −80°C and transported on dry ice for testing. Aliquots used in this study were subjected to one freeze/thaw cycle in the process of aliquotting upon receipt at NHRC, and one further freeze/thaw cycle when aliquotted for shipment to Travis AFB. These samples were otherwise unprocessed, collected, and transported under College of American Pathologists (CAP) accredited diagnostic protocols.

### Specimen processing

Nasal wash samples were collected in approximately 2 mls of saline and nucleic acid extraction was performed at room temperature prior to freezing of the remaining aliquots. From each sample, 400 µl was aliquoted into 2 ml microcentrifuge tubes for automated DNA extraction. Extractions were performed using the MagNA Pure Compact Nucleic Acid Isolation Kit I (Roche Diagnostics, Mannheim Germany, MagNA Pure Kit 03 730 964 001) with the MagNA Pure Compact instrument by Roche, Mannheim, Germany. The MagNA Pure Compact instrument was programmed to extract a 400 µl sample and elute 200 µl of purified DNA (Ver. 1.0; software version 1.1 DNA_Bacteria purification protocol) for nasal wash samples.

Throat swabs and cultured isolates were processed in a BSL-2 hood. Nucleic acid was extracted from 175 µL aliquots using the MagNA Pure LC DNA Isolation Kit I (Roche, Indianapolis, IN, USA) according to the manufacturers' recommendations for the MagNA Pure LC automated nucleic acid extraction system. The following adenovirus strains were used as controls for reactivity with our HAdV-14 assay: Adenovirus strains HAdV-14 (VR-15), -3 (VR-3), -4 (VR-1572), -7 (VR-7), -11 (VR-12), -21 (VR-256), Haemophilus influenza, Influenza A virus (ATCC VR-96), Human rhinovirus 14 (ATCC VR-284), Human parainfluenza virus 2 (ATCC VR-92), Human respiratory syncytial virus (ATCC VR-26), *Chlamydophila pneumonia* (ATCC 53592), *Escherichia Coli*, *Klebsiella pneumonia* (ATCC 13883), *Pseudomonas aeruginosa* (ATCC 97), *Mycoplasma pneumonia*, and *Legionella Pneumophila* (ATCC 33152) were acquired from the American Type Culture Collection (ATCC; Manassas, VA, USA).

### Conventional PCR testing

The primers used to amplify HAdV-14 by conventional PCR were Ad14F 5′-AAATGCTAATCTTGGACAGCAGTC-3′ and Ad14R 5′- AGCCGTCCAGTGGAAAACAGTAGT-3′. Reaction mixtures for conventional PCR performed at the Naval Health Research Center contained 1 µL of Q-Solution (QIAGEN), 1 µL PCR buffer (Promega, Madison, Wis.), 0.6 µM concentration of each primer (Integrated DNA Technologies), 0.8 mM concentration of each deoxynucleoside triphosphate (Promega), 3 mM concentration of MgCl_2_ (Promega), 1.25 U of *Taq* polymerase (Promega), and 2 µLs of extracted sample in 25-µL aqueous reactions. Reaction conditions were as follows: initial denaturation at 95°C for 5 min, followed by 35 cycles of denaturation at 95°C for 1 min, annealing at 53°C for 1 min, and extension at 72°C for 1 min. Cycling conditions included a 10 min final extension at 72°C and a final hold at 4°C.

### Quantitative real-time PCR

The primers used to generate the HAdV-14 amplicon were prAd14-hex2F, 5′-GGGTTGAAACTACTGAAGAACG-3′ and prAd14-hex2R, 5′-CATCTGTATCAGTCCACGATT-3′. The TaqMan probe used to detect the hexon gene have the following sequence: Ad14-TaqMan1, [6∼FAM]AGCTACATACACTTTTGGCAATGCC[Tamra∼Q]. Experiments were performed on a Joint Biological Agent Identification and Diagnostic System (J.B.A.I.D.S) and the LightCycler 2.0 (Roche). The J.B.A.I.D.S. thermocycler was conceived and developed to rapidly identify biological warfare agents and other pathogens of concern for the U.S. Military. For real-time J.B.A.I.D.S. PCR, cycling was carried out in a J.B.A.I.D.S. real-time thermocycler (Idaho Technologies, UT, USA) using 1 µl (∼1.8 µL of throat swab) of extracted DNA in 10 µl of TaqMan Universal PCR Master Mix (Roche), containing 5 mM MgCl_2_ and 400 nM forward and reverse primers. Reaction conditions were as follows: initial denaturation at 95°C for 15 min, followed by 45 cycles of denaturation at 95°C for 1 s, annealing at 60°C for 15 s, and extension at 72°C for 5 s. The progress of real-time fluorescent PCR was monitored at 530 nm. To establish external standard curves for the quantification of HAdV-14, genomic DNA from the prototype de Wit strain (HAdV-14p) were diluted in a 10-fold series (10^2^ to 10^7^ copies per reaction) and analyzed with the new assay. The samples that defined the standard curve were performed in triplicate. The crossing point (CP) for 10^7^ was 17.4 and the CP for 100 copies was 37.3. Triplicate samples were used to generate standard curve experiments. Standard curves were repeated twice and we saw similar results. Samples were considered positive if they had a crossing point less than 40 (Fig. 1).

### IRB approval

Informed consent was obtained in writing. This study was approved by the Wilford Hall Institutional Review Board as well as the 60th Medical Group Institutional Review Board under Clinical Investigation No. FDG20080029E.
